# Peripartum depression and infant care, sleep and growth

**DOI:** 10.1038/s41598-019-46563-4

**Published:** 2019-07-15

**Authors:** Sachiko Iwata, Masahiro Kinoshita, Fumie Fujita, Kennosuke Tsuda, Mitsuaki Unno, Takashi Horinouchi, Seiichi Morokuma, Shinji Saitoh, Osuke Iwata

**Affiliations:** 1 Center for Human Development and Family Science, Department of Pediatrics and Neonatology, Nagoya City University Graduate School of Medical Sciences, Nagoya, Japan; 2 Centre for Developmental and Cognitive Neuroscience, Kurume University School of Medicine, Kurume, Japan; 3 Department of Obstetrics and Gynaecology, Kurume University School of Medicine, Kurume, Japan; 4 Department of Health Sciences, Graduate School of Medical Sciences, Kyushu University, Fukuoka, Japan

**Keywords:** Sleep, Epidemiology, Risk factors

## Abstract

Peripartum depression is a common, serious complication in mothers. To assess the influence of infant care, sleep and growth on the risk of peripartum depression, 1,271 mothers of healthy one-month-old infants completed comprehensive questionnaires including the Edinburgh Postnatal Depression Scale. Relationships between high depression scores and variables related to infants’ care, sleep and growth were assessed adjusting for other variables. High depression scores were found in 233 mothers, which were associated with variables related to infants’ care (poor satisfaction with infant care support, p < 0.001; mothers’ passive response to night-time fussing, p = 0.001), sleep (dim bedroom lighting, p < 0.05; short sleep duration, p < 0.05) and growth (poor weight gain, p < 0.05), as well as maternal variables (primiparas, p < 0.001; poor income satisfaction, p < 0.001; poor sleep satisfaction, p < 0.001; daytime sleepiness, p = 0.001). Considering the observed association between high depression scores and infants’ care, sleep and growth, a multidisciplinary approach accounting for infant care would be required to prevent peripartum depression.

## Introduction

According to a systematic review conducted in the United States, approximately 20% of women have a major depressive episode during the first 3 months postpartum^1^, which is considerably higher than <10% of women in the general population^2^. Peripartum depression (PD) is associated with long-term, compromising effects on the mother-infant relationship and development of infants^3,4^. Researchers investigated the potential dependence of PD on various factors, such as ethnicity, family history of depression, socioeconomic status, type of feeding and maternal oxytocin levels^5,6^. In addition to maternal variables, several studies investigated the role of factors related to infant care. For example, serious sleeping and crying problems in young infants are associated with a two-fold increase in maternal depression and anxiety^7^, and inappropriate social infant care support is associated with an elevated incidence of PD^8^. Vigod *et al*. described that delivery of low-birth-weight infants and their admission to the neonatal intensive care unit are associated with an increased risk of maternal PD, suggesting that growth and health status of infants might also be associated with the risk of PD^9^. These findings suggest that strategies to predict and prevent PD may need to account for variables rooted in various aspects of infant care, as well as maternal and socioeconomic variables. Thus far, however, few comprehensive studies have addressed the role of factors related to the infant care, sleep and growth in the development of PD in mothers with healthy infants.

The aim of this study was to assess whether infant care, sleep and growth are associated with the risk of PD even when adjusted for other known and unknown independent variables in a large cohort of mothers with healthy young infants.

## Results

Data are presented as mean ± standard deviation, number (%) or median (interquartile range). Clinical backgrounds of the study population have been published previously^10^. Of the questionnaires collected from 1,302 participating mothers, data from 31 mothers were excluded due to excessive missing data. The 1,271 mothers in the final study cohort were 31.9 ± 4.6 years old (Table 1). The highest education was high school or college in 1,228 (96.6%) mothers; 1,164 (91.6%) mothers were fully or generally satisfied with their family income; the typical night-time sleep duration of the mothers was 5.4 ± 1.5 h, with 619 (48.7%) mothers dissatisfied with their sleep (Table 1). High Edinburgh Postnatal Depression Scale (EPDS) scores (≥9) were noted in 233 (18.3%) mothers, whereas very high EPDS scores (≥12) were found in 115 (9.0%) mothers. The infants were 39.5 ± 1.2 weeks gestation and 3091 ± 374 g at birth, whose body weight gain was 44.1 ± 10.4 g/day. The night-time and daily sleep durations of the infants were 8.3 ± 1.8 h and 15.8 ± 2.8 h, respectively.

**Table 1:** Clinical backgrounds of participating mothers and infants.

Variables		n (%)
**Infant variables**		
Body weight at birth (g)		3091 ± 374
Postnatal age (day)		33.9 ± 5.8
Body weight gain (g/day)		44.1 ± 10.4
Light-off time for infants	Regularly ≤ 21:00 h	437 (34.4%)
	Regularly > 21:00 h	549 (43.2%)
	Irregular	285 (22.4%)
Daytime sleep duration (h)		7.5 ± 1.9
Night-time sleep duration (h)		8.3 ± 1.8
Room brightness	Bright	942 (74.1%)
	Dim	329 (25.9%)
Type of milk	Exclusive breast milk	663 (52.2%)
	Mainly breast milk	342 (26.9%)
	Mainly formula milk	266 (20.9%)
**Maternal variables**		
Age (year)		31.9 ± 4.6
Highest education	Junior high school	43 (3.4%)
	High school	297 (23.4%)
	College	931 (73.2%)
Satisfaction with income	Fully satisfied	206 (16.2%)
	Generally satisfied	958 (75.4%)
	Not satisfied	107 (8.4%)
Gestational age at delivery (week)		39.5 ± 1.2
Parity	1	540 (42.5%)
	2	474 (37.3%)
	≥3	257 (20.2%)
Delivery mode	Vaginal	1074 (84.5%)
	Caesarean (elective)	142 (11.2%)
	Caesarean (emergency)	55 (4.3%)
Night-time sleep duration (h)		5.4 ± 1.5
Satisfaction with sleep	Satisfied	185 (14.6%)
	Mildly dissatisfied	467 (36.7%)
	Dissatisfied	619 (48.7%)
Epworth sleepiness scale		3 (5–7)
Edinburgh Postnatal Depression Scale		4 (0–10)

Values are shown as number (%), mean ± standard deviation or median (interquartile range).

Findings from univariate analysis: variables associated with infant care, sleep and growth (Table 2).

**Table 2:** Independent variables of high EPDS scores identified by univariate analysis: variables associated with infant care, sleep and growth.

Variables		n	OR	95% CI		p
				Lower	Upper	
Gestational age at delivery (week)			1.076	0.952	1.216	0.239
Body weight at birth (g)			1.000	1.000	1.000	0.758
Postnatal age (day)			1.008	0.982	1.036	0.538
Body weight gain (g/day)			0.974	0.961	0.987	<0.001
**Infant sleep and environment**						
Light-off time for infants	Regularly ≤ 21:00 h	437	1	Reference		
	Regularly > 21:00 h	549	1.126	0.801	1.584	0.494
	Irregular	285	1.784	1.228	2.592	0.002
Daytime sleep duration (h)	9≤	352	1	Reference		
	6 ≤ sleep duration < 9	726	1.466	1.004	2.140	0.048
	<6	193	1.880	1.170	3.020	0.009
Night-time sleep duration (h)	10≤	362	1	Reference		
	7.5 ≤ sleep duration <10	575	1.476	1.015	2.146	0.041
	<7.5	334	2.152	1.433	3.231	<0.001
Daily sleep duration (h)	18 ≤	350	1	Reference		
	14 ≤ sleep duration <18	561	2.279	1.476	3.518	<0.001
	<14	360	2.489	1.612	3.843	<0.001
Room brightness	Bright	942	1	Reference		
	Dim	329	1.411	1.031	1.929	0.031
**Social infant care support and child-rearing style**						
Additional caregivers	None	108	1	Reference		
	1	521	2.194	1.161	4.147	0.016
	≥2	642	1.633	0.865	3.083	0.131
Satisfaction with support	No support	108	1	Reference		
	Fully satisfied	466	1.242	0.644	2.396	0.518
	Generally satisfied	631	2.090	1.112	3.930	0.022
	Not satisfied	66	5.776	2.630	12.686	<0.001
Type of milk	Exclusive breast milk	663	1	Reference		
	Mainly breast milk	342	1.305	0.927	1.838	0.127
	Mainly formula milk	266	1.682	1.181	2.397	0.004
Response to night-time fussing	Intermediate (rock briefly)	453	1	Reference		
	Aggressive (rock until calm)	432	1.348	0.934	1.946	0.111
	Passive (observe)	386	2.161	1.516	3.081	<0.001
Confidence in child-rearing	Confident	331	1	Reference		
	Generally confident	669	2.575	1.520	4.362	<0.001
	Anxious	271	15.738	9.232	26.831	<0.001

Elevated EPDS scores were associated with smaller body weight gain of infants (p < 0.001), dim lighting of infant’s bedroom (p = 0.031 for ‘dim’ compared with ‘bright’), irregular light-off time (p = 0.002 for ‘irregular’ compared with ‘regularly early’), shorter daytime sleep duration (p = 0.009 for ‘short’ and 0.048 for ‘medium’; compared with ‘long’), shorter night-time sleep duration (p < 0.001 for ‘short’ and p = 0.041 for ‘medium’; compared with ‘long’), and shorter daily sleep duration (p < 0.001 for both ‘short’ and ‘medium’ compared with ‘long’). For variables associated with infant care support and child-rearing style, the presence of an additional caregivers (p = 0.016 for ‘one’ compared with ‘no’ extra caregiver), poor satisfaction with social infant care support (p = 0.022 for ‘generally satisfied’ and p < 0.001 for ‘not satisfied’; compared with ‘no support’), formula-milk-based feeding (p = 0.004 for ‘mainly formula milk’ compared with ‘exclusive breastfeeding’), passive response of mothers to night-time fussing (p < 0.001 for ‘observe’ without dandling compared with ‘rock briefly’), and poor confidence with child rearing (p < 0.001 for mothers of both ‘anxious’ and ‘generally confident’; compared with ‘confident’) were associated with high EPDS scores.

### Findings from univariate analysis: maternal variables

Higher maternal age (p = 0.032 for ≥35 years old compared with 29≤ and <35 years old) and dissatisfaction with family income (p < 0.001 for ‘not satisfied’ and p = 0.003 for ‘generally satisfied’; compared with ‘fully satisfied’), primipara status (p < 0.001 for primiparas compared with both parity 2 and ≥3) and vaginal delivery (p = 0.030 for vaginal delivery compared with elective caesarean delivery) were associated with high EPDS scores (Table 3). Of variables associated with maternal sleep status during the last 7 days of the baby check, irregular bedtime (p < 0.001 for ‘irregular’ and p = 0.027 for ‘regularly late’; compared with ‘regularly early’), shorter night-time sleep duration (p < 0.001 for ‘short’ and p = 0.043 for ‘medium’; compared with ‘long’), more frequent night-time wakefulness (p < 0.001), poor satisfaction with sleep (p < 0.001 for both ‘dissatisfied’ and ‘mildly satisfied’; compared with ‘satisfied’), and greater Epworth Sleepiness Scale (ESS) scores (p < 0.001) were associated with high EPDS scores (See Supplementary Table 1 for the variables in relation to maternal sleep during the last month of pregnancy).

**Table 3:** Independent variables of high EPDS scores identified by univariate analysis: maternal variables.

Variables		n	OR	95% CI		p
				Lower	Upper	
Age (year)	<29	289	1.309	0.910	1.884	0.147
	29 ≤ age <35	595	1	Reference		
	35 ≤	387	1.433	1.032	1.990	0.032
Highest education	Junior high school	43	1	Reference		
	High school	297	0.983	0.448	2.159	0.966
	College	931	0.800	0.377	1.697	0.561
Satisfaction with income	Fully satisfied	206	1	Reference		
	Generally satisfied	958	2.121	1.301	3.459	0.003
	Not satisfied	107	4.061	2.181	7.559	<0.001
Gestational age at delivery (week)			1.076	0.952	1.216	0.239
Parity	1	540	1	Reference		
	2	474	0.477	0.344	0.661	<0.001
	≥3	257	0.400	0.259	0.618	<0.001
Delivery mode	Vaginal	1074	1	Reference		
	Caesarean (elective)	142	0.547	0.318	0.943	0.030
	Caesarean (emergency)	55	1.185	0.615	2.283	0.614
**Sleep status within 7 days**						
Bedtime	Regularly ≤ 23:00 h	453	1	Reference		
	Regularly > 23:00 h	208	1.782	1.067	2.975	0.027
	Irregular	610	3.088	2.126	4.485	<0.001
Wake time	8:00 h≤	360	1	Reference		
	6:00 h ≤ wake time < 8:00 h	555	0.818	0.582	1.149	0.246
	<6:00 h	356	0.766	0.525	1.119	0.168
Night-time sleep duration (h)	6≤	571	1	Reference		
	4 ≤ sleep duration < 6	395	1.467	1.012	2.127	0.043
	<4	305	2.994	2.111	4.247	<0.001
Wakefulness episodes			1.318	1.138	1.526	<0.001
Satisfaction with sleep	Satisfied	185	1	Reference		
	Mildly dissatisfied	467	5.573	2.637	11.776	<0.001
	Dissatisfied	619	5.667	2.713	11.838	<0.001
Epworth sleepiness scale ≥ 11		84	2.725	1.701	4.367	<0.001

See Supplementary Table 1 for the analysis using maternal sleep status before delivery.

Abbreviations: CI, confidence interval. EPDS, Edinburgh Postnatal Depression Scale. OR, odds ratio.

### Multivariate analysis

In the multivariate analysis, high EPDS scores were associated with poor satisfaction with family income (p < 0.001 for ‘unsatisfied’ and p = 0.017 for ‘generally satisfied’; compared with ‘fully satisfied’), primipara status (p = 0.001 and p < 0.001 for primiparas; compared with parity 2 and ≥ 3, respectively), poor satisfaction with social infant care support (p = 0.041 for ‘generally satisfied’ and p < 0.001 for ‘not satisfied’; compared with ‘no social infant care support’), dim room lighting (p = 0.024 for ‘dim’ compared with ‘bright’), passive response of mothers to night-time fussing (p = 0.001 for ‘observation’ without dandling compared with ‘dandling briefly’), lower weight gain of infants (p = 0.044), shorter daily sleep duration of infants (p = 0.045 for ‘short’ and p = 0.049 for ‘medium’; compared with ‘long’), poor maternal satisfaction with sleep (p < 0.001 for both ‘mildly dissatisfied’ and ‘dissatisfied’; compared with ‘satisfied’), and greater postpartum ESS scores (p = 0.001) (Table 4).

**Table 4:** Independent variables of high EPDS scores identified by multivariate analysis.

Variables		OR	95% CI		p
			Lower	Upper	
**Infant care, sleep and growth**					
Body weight gain (g/d)		0.981	0.964	0.999	0.044
Room brightness	Bright	1	Reference		
	Dim	1.490	1.055	2.104	0.024
Daily sleep duration (h)	18≤	1	Reference		
	14 ≤ sleep duration < 18	1.758	1.003	3.080	0.049
	<14	1.766	1.012	3.083	0.045
**Maternal satisfaction with social infant care support**					
	No support	1	Reference		
	Fully satisfied	1.178	0.578	2.401	0.652
	Generally satisfied	2.034	1.031	4.012	0.041
	Not satisfied	6.896	2.917	16.306	<0.001
**Maternal response to night-time fussing**					
	Intermediate (rock briefly)	1	Reference		
	Aggressive (rock until calm)	1.333	0.900	1.975	0.152
	Passive (observe)	1.887	1.286	2.770	0.001
**Maternal variables**					
Satisfaction with income	Fully satisfied	1	Reference		
	Generally satisfied	1.873	1.117	3.141	0.017
	Not satisfied	3.671	1.893	7.122	<0.001
Parity	1	1	Reference		
	2	0.522	0.363	0.751	0.001
	≥3	0.377	0.231	0.615	<0.001
Satisfaction with sleep	Satisfied	1	Reference		
	Mildly dissatisfied	4.639	2.132	10.093	<0.001
	Dissatisfied	4.834	2.260	10.341	<0.001
Epworth sleepiness scale ≥ 11		2.362	1.410	3.959	0.001

Abbreviations: CI, confidence interval. EPDS, Edinburgh Postnatal Depression Scale. OR, odds ratio.

## Discussion

Using a comprehensive dataset from a survey of mothers of 1-month-old infants, infant care, sleep and growth were identified as potential independent variables of PD even after adjustment for the influence of known independent variables. These findings suggest that, for the prediction, prevention, early diagnosis and efficient treatment of PD, multifactorial approach would be required focusing on both maternal and non-maternal variables, especially on those related to the care, sleep and growth of infants.

### Peripartum depression and sleep of mothers and infants

Infants’ sleep duration start aggregating in the night-time approximately one month after birth; however, the timing and progress vary between infants^11^. A delay in this process may increase the anxiety of mothers, rendering them susceptible to psychiatric conditions. Our data suggest the dependence of maternal PD on the schedule, quantity, and environment of their infants’ sleep, as well as their own sleep quality and quantity. Using the same dataset, we have previously demonstrated that maternal night-time sleep duration is tightly coupled with that of their infants^10^. These findings suggest the importance of providing good night-time sleep to infants in order to secure good maternal sleep and ultimately reduce the risk of maternal PD. We have previously shown that punctual light-off time is associated with longer night-time sleep duration in infants^10^. Univariate analysis in the current study suggested that punctual light-off time for infants and punctual bedtime for mothers are associated with a lower risk of maternal PD. Regular, and possibly early, light-off time for infants might promote a swift acquisition of mature sleep patterns, potentially leading to better maternal sleep and lower incidence of PD.

### Peripartum depression and infant care

Pathological clinical conditions of newborn infants, such as preterm delivery and hospitalisation of infants at neonatal intensive care centres, have been recognised as prominent risk variables of PD^9^. Our study suggested that, even after delivery of a healthy infant, relatively poor infant growth might be the risk of PD. Because infant weight is one of the few visible markers of ‘successful infant care’ for mothers, poor weight gain might easily enhance mothers’ anxiety. Univariate analysis in our study suggested that, in addition to the infant growth, mothers’ confidence and anxiety regarding infant care was associated with high EPDS scores. These findings highlight the importance of attending to mothers’ psychological characteristics to predict and prevent PD. To ameliorate the excessive pressure on mothers, it is important to provide appropriate social infant care support^8^. In the current study, the risk of PD was paradoxically dependent on the type and quality of social infant care support. While dissatisfaction with social infant care support was associated with high EPDS scores, mothers who were caring for their infants on their own showed the lowest risk of PD, suggesting that satisfaction with social infant care support is not necessarily dose-dependent, but is affected by mothers’ psychological characteristics.

With respect to the type of feeding, our findings from the univariate analysis were consistent with previous studies showing the benefits of breastfeeding in reducing the risk of PD^12,13^. Breastfeeding mothers are exempted from additional night-time tasks required to prepare the formula milk, allowing them longer night-time sleep duration. Increased serum oxytocin levels of breastfeeding mothers may also influence the threshold for developing PD^14^. Although our data suggest the benefit of exclusive breastfeeding, the supplemental use of formula milk did not increase the risk of PD. Given that failure to establish breastfeeding is associated with a significant increase in the risk of PD^15^, encouragement of breastfeeding should be accompanied by sufficient support to minimise maternal psychological pressure.

### Other independent variables of peripartum depression

Consistent to previous studies, which demonstrated the association between maternal socio-economic status and the development of PD^5,6^, we confirmed that poor satisfaction of mothers with their family income was associated with high EPDS scores. Dependence of EPDS scores on delivery mode and parity was also suggested. However, unlike a previous study, which observed an elevated risk of PD following caesarean delivery^16^, our data in the univariate analysis suggested the association between elective caesarean delivery and lower risk of PD. Background characteristics associated with the indication of caesarean delivery might be associated with the paradoxical impact of caesarean delivery to the risk of PD. In our study, the majority of women who underwent caesarean delivery were multipara mothers, however, no further data were available regarding the indication of caesarean delivery. There currently is no large-scale survey, which assessed the indication of caesarean delivery in Japanese women. In Japan, the rate of elective caesarean delivery is 11.0%, which is lower than in other developed countries^17^. Considering that this delivery mode is covered by the national insurance, it is unlikely that socio-economic status affected the decision of the delivery mode in our study cohort. Future studies need to address the impact of caesarean delivery on the risk of PD.

### Strength and limitation of the study

The development of PD is dependent on factors rooted in various aspects of maternal life, such as socio-economic status, pregnancy plan, problems with family and spouse, history of mental illness in the family, and social support^5,6^, suggesting that maternal background is insufficient to fully explain the mechanism of PD. By incorporating various background variables associated with maternal life during and after delivery, we have demonstrated that variables associated with infant care, sleep and growth are key factors to understand the mechanism of maternal PD. However, considering that clinical variables of mothers, infants, and other family members are often inter-correlated with each other (such as lifestyle and sleep schedule), it remains uncertain whether variables other than those related to maternal background are directly associated with PD, or merely via the inter-correlation between the variables. As previously described, our study was conducted by recruiting typical Japanese mothers in terms of age, academic background, and other socio-economic variables^18^. Second, we used a relatively low cut-off value of EPDS ≥ 9 to represent increased risks of PD^19–21^. Based on the high-enough sensitivity and specificity of this threshold to predict PD in Japanese women (82% and 95%, respectively), it was speculated that Japanese women are too reluctant to express their feelings and emotions when they are depressed^21^. Therefore, when generalising our findings to mothers in other parts of the world, careful consideration is required for cultural and traditional factors.

## Conclusions

In addition to established maternal factors associated with PD, our survey with mothers of healthy 1-month-old infants highlighted that independent variables rooted in infant care, growth and sleep, were associated with the risk of maternal PD even when adjusted for known independent variables. A comprehensive, multidisciplinary approach is required to predict, prevent, identify, and treat maternal PD. Future studies need to address whether a strategy, which incorporate infant care, growth and sleep, improve prediction and prevention of PD, and thus help minimise the adverse influence of PD on the mother-child relationship.

## Methods

### Ethics statement

This study was conducted in compliance with the Declaration of Helsinki, and with the approval of the ethics committees of Kurume University School of Medicine and Kyushu University School of Medicine. Informed consent was obtained from all participating mothers, who answered the survey.

### Study population

Data collection was performed with the participation of two tertiary perinatal centres (Kurume University Hospital and Kyushu University Hospital) and three private obstetrics hospitals (Izumi Ladies’ Clinic, Fukuoka Birth Clinic, and Sato Women’s Hospital) in Fukuoka, Japan. Preliminary data on the photoperiod and sleep patterns of infants and their mothers were reported previously, and data collection methods were described in detail^10^. Briefly, mothers of newborn infants were asked to complete a questionnaire at the time of the 1-month baby check between December 2014 and November 2015.

### Questionnaires

Information was collected using the originally developed Sleep Questionnaire for Mothers of Newborn Infants^10^, EPDS^20–22^ and ESS^23,24^. Our original sleep questionnaire comprised 45 questions in relation to mothers and their family, birth of infants, social infant care support, infants’ sleep pattern and environment, and mothers’ sleep condition^10^. For the current study, 30 questions were carefully chosen to represent the variables of interest; relatively a wide range of variables (birth information, child-rearing style, growth and sleep of infants, and maternal sleep status before and after delivery, and ESS scores after delivery) were considered to assess the potential dependence of high EPDS scores on infant care, sleep and growth with adjustment for known independent factors of PD and other factors associated with various aspects of maternal life. The ESS assesses sleepiness while engaged in eight different activities (score 0–24), with higher scores indicating higher daytime sleepiness^23^. The EPDS is a 10-item self-report scale specifically designed to screen for postpartum depressive symptoms (score 0–30), with higher scores indicated higher levels of depressive symptomatology. For the Japanese version of EPDS, a relatively low cut-off score of 8/9 is recommended for the screening of PD^19^.

### Data analysis

Questionnaires with more than 20% missing data without relevant reasons were excluded from the analysis^25^. To minimise biases derived from missing data, multiple imputation of the variable was performed (n = 5 imputations) based on the correlation between variables with missing values and other participant characteristics using SPSS ver. 21.0 (IBM, Armonk, NY, U.S.A.). A univariate logistic regression model was used to evaluate the association of potential independent variables with EPDS scores ≥9. Continuous variables with normal distribution were categorised into 2–3 levels using quartiles or clinically relevant thresholds when appropriate. P-values for the univariate analysis were not corrected for multiple comparisons because of the exploratory nature of the analysis. However, to avoid type-1 errors, p-values between 0.01 and 0.05 were regarded as chance level. A multivariate logistic regression analysis was performed to identify the final model to explain EPDS scores ≥9. For the multivariate model, mandatory variables (e.g. satisfaction with infant care support, body weight gain, daily sleep duration of infants, maternal satisfaction with sleep, and maternal satisfaction with family income) were chosen based on our hypothesis driven by previous studies, whereas additional variables were chosen based on clinical relevance, collinearity and the univariate analysis results.

## Supplementary information

Supplementary Information
