# Supplementary material for: Peripartum depression and infant care, sleep and growth
Source: Sci Rep. 2019 Jul 15;9:10186. doi: 10.1038/s41598-019-46563-4 (PMC6629993; doi:10.1038/s41598-019-46563-4)
Supplement: Supplementary file 1 — Supplementary Information [file 41598_2019_46563_MOESM1_ESM.docx]

**Online Supplemental Information**

**Title**

Peripartum depression and infant care, sleep and growth

**Authors**

Sachiko Iwata^1^, Masahiro Kinoshita^2^, Fumie Fujita^2^, Kennosuke Tsuda^1^, Mitsuaki Unno^2^, Takashi Horinouchi^3^, Seiichi Morokuma^4^, Shinji Saitoh^1^, and Osuke Iwata^12^.

**Affiliations**

^1^Center for Human Development and Family Science, Department of Neonatology and Pediatrics, Nagoya City University Graduate School of Medical Sciences, Aichi, Japan.

^2^Centre for Developmental & Cognitive Neuroscience, Kurume University School of Medicine, Kurume, Fukuoka, Japan.

^3^Dept of Obstetrics and Gynaecology, Kurume University School of Medicine, Fukuoka, Japan.

^4^Dept of Obstetrics and Gynecology, Kyushu University School of Medicine, Fukuoka, Japan.

**Correspondence to:**

Dr Osuke Iwata

Center for Human Development and Family Science, Department of Neonatology and Pediatrics, Nagoya City University Graduate School of Medical Sciences, 1 Kawasumi, Mizuho-Cho, Mizuho-ku, Nagoya, 467-8601 Japan.

E-mail: o.iwata@med.nagoya-cu.ac.jp

Tel: +81 52 853-8246 Fax: +81 52 842-3449

Online Supplementary Table 1: Independent variables of high EPDS scores: univariate analysis for maternal sleep status during the last month of pregnancy

| Variables |  | n | OR | 95% CI | | p |
| --- | --- | --- | --- | --- | --- | --- |
|  |  |  |  | Lower | Upper |  |
| Bedtime | Regularly ≤ 23:00 h | 645 | 1 | Reference | |  |
|  | Regularly > 23:00 h | 284 | 1.092 | 0.756 | 1.579 | 0.639 |
|  | Irregular | 342 | 1.232 | 0.875 | 1.734 | 0.232 |
| Wake time | 7:30 h ≤ | 304 | 1 | Reference | |  |
|  | 6:00 h ≤ wake time <7:30 h | 639 | 1.015 | 0.708 | 1.456 | 0.935 |
|  | 6:00 h  6:00 h ≤ wake time <7:30 h  7:30 h ≤ (more75%tile) | 328 | 1.250 | 0.837 | 1.867 | 0.276 |
| Night-time sleep duration (h) | 8 ≤ | 342 | 1 | Reference | |  |
|  | 6 ≤ sleep period < 8 | 375 | 1.159 | 0.761 | 1.766 | 0.490 |
|  | < 6 | 554 | 1.44 | 0.992 | 2.090 | 0.055 |
| Wakefulness episodes |  |  | 1.180 | 1.050 | 1.326 | 0.005 |
| Satisfaction with sleep | Satisfied | 458 | 1 | Reference | |  |
|  | Mildly dissatisfied | 320 | 1.387 | 0.951 | 2.024 | 0.089 |
|  | Dissatisfied | 493 | 1.435 | 1.024 | 2.013 | 0.036 |

More frequent night-time wakefulness (p=0.005) and poor satisfaction with sleep (p=0.036 for ‘dissatisfied’; compared with ‘satisfied’) were associated with high EPDS scores).

Abbreviations: CI, confidence interval. EPDS, Edinburgh Postnatal Depression Scale. OR, odds ratio.
